# Factors influencing Australian podiatrists’ behavioural intentions to adopt a smart insole into clinical practice: a mixed methods study

**DOI:** 10.1186/s13047-020-00396-x

**Published:** 2020-06-01

**Authors:** Emma M. Macdonald, Byron M. Perrin, Michael I. C. Kingsley

**Affiliations:** 1 Holsworth Research Initiative, La Trobe Rural Health School, La Trobe University, Bendigo, Australia; 2 Diabetes Centre, Goulburn Valley Health, Shepparton, Australia; 3 Department of Exercise Sciences, University of Auckland, Auckland, New Zealand

**Keywords:** Podiatrist, Diabetes mellitus, Foot ulceration, Peripheral neuropathy, Smart insole, Wearable technology

## Abstract

**Background:**

Diabetes is the leading cause of lower limb amputation in Australia, costing the Australian health care system an estimated A$1.6 billion annually. Podiatrists are the primary foot health care provider in Australia. Research suggests that health professional attitudes can impact patient utilisation of e-health technologies, such as wearable foot monitoring devices aimed at preventing foot ulceration. The aim of this study was to explore factors that impact the intentions of Australian podiatrists to adopt smart insole foot monitoring technology.

**Methods:**

A mixed methods explanatory sequential design was undertaken. One hundred and eleven Australian podiatrists completed an online version of the validated Unified Theory of Acceptance and Use of Technology (UTAUT) questionnaire. Multiple regression analysis was used to determine the strongest predictive model of podiatrists’ behavioural intention to adopt technology. Additionally, two focus groups were conducted, and thematic analysis was performed to explore podiatrists’ perceived barriers and enablers to smart insole adoption.

**Results:**

One hundred and eleven Australian podiatrists completed the online UTAUT questionnaire. The majority of respondents practiced in the private sector (58.6%) and were female (50.5%), with Victoria the most common practice location (39.6%). Significant positive correlations existed between behavioural intention and six psychosocial domains including performance expectancy (*r* = 0.64, *p* < 0.001), effort expectancy (*r* = 0.47, *p* < 0.001), attitude (*r* = 0.55, *p* < 0.001), social influence (*r* = 0.45, *p* < 0.001), facilitating conditions (*r* = 0.36, *p* < 0.001), and self-efficacy (*r* = 0.30, *p* < 0.002). Multiple regression analysis determined that performance expectancy alone was most predictive of behavioural intention to adopt a smart insole into clinical practice (adjusted R^2^ = 42%, *p* < 0.001). Qualitative analyses revealed that podiatrists believed that the insole would increase patient knowledge, engagement and self-efficacy. However, concerns were raised about cost, footwear issues and the device’s utility with elderly and remote populations.

**Conclusions:**

Performance expectancy was the most important psychosocial factor predicting the intentions of Australian podiatrists to adopt smart insole foot monitoring technologies. While Australian podiatrists are open to adopting smart insoles into clinical practice, evidence of the device’s efficacy is a precursor to adoption. Other perceived barriers to adoption including device cost, compatibility with off-loading, footwear issues and patient age also need to be addressed prior to implementation and clinical adoption.

## Background

Diabetes-related foot disease (DFD) is the leading cause of lower limb amputation [1–4] and costs an estimated A$1.6 billion annually [2]. DFD commonly consists of ulceration, infection, ischaemia or neuro-arthropathy [2–4]. Following initial foot ulcer healing, 40% of people re-ulcerate within the first year and up to 60% will re-ulcerate within 3 years [3]. Over the last decade, Australia has had one of the highest rates of diabetes related amputation in the developed world [1], with rurality and remoteness being associated with an even higher risk [5]. In order to reduce the incidence of avoidable ulceration and amputations peak bodies including the International Diabetes Federation, International Working Group on the Diabetic Foot, and more locally, Diabetic Foot Australia (DFA), have developed evidence-based guidelines and strategies to inform health professionals and policy-makers [4, 6–9]. These strategies include earlier identification of those at risk and government funding to improve access to preventative services [4, 6], such as appropriate footwear and off-loading [6]. Acknowledging wide variation in climate and cultural attitudes towards footwear and off-loading, DFA have opted for a nationally consistent presentation of recommendations to patients [6]. In Australia, podiatrists are often the primary foot health care provider for people with diabetes, and are involved in providing many of the recommended DFD assessments and therapies [6].

In the context of the global effort to reduce primary and recurrent ulceration rates there has been increased interest in developing new, wearable forms of continuous foot monitoring devices. These devices aim to empower the wearer to make real-time modifications to behaviour, in order to prevent both primary and secondary ulcerations [10]. A range of sensor and material types are being developed [11] including smart socks [12–14], insoles [15, 16] and mats [17], and our recent work indicates that people with diabetes have a positive attitude towards adopting such technologies [18]. However, in order for these types of devices to realise their potential they must be adopted by health professionals, such as podiatrists, who are in a position to make foot monitoring recommendations that influence their patient’s attitudes and behaviours towards adopting these technologies [19].

A systematic review of factors affecting patient adoption and utilisation of technology to support diabetes management identified eleven studies that highlighted the influence that health professionals had on their patients’ willingness to adopt and use a technology. The attitudes of health professionals in these studies directly impacted their patients’ technology utilisation. When health professionals encouraged and promoted a technology to their patients, the patients were more likely to adopt the technology. Furthermore, when health professionals engaged with data generated by the technology over the medium term, patients were more likely to maintain utilisation [19]. Therefore, understanding the attitudes of podiatrists treating people with diabetes-related neuropathy toward their patient’s use of a ‘smart’ insole is important if this emerging technology type is to be widely adopted to prevent neuropathic foot ulceration.

This study aims to identify and explore the personal and professional factors that influence podiatrists’ intention to adopt smart insole technology into their clinical practice.

## Methods

### Study design

A mixed methods explanatory sequential design was undertaken. The researchers used the definition provided by Morse [20] requiring that mixed methods research use more than one methodological approach for the same investigation and that the analysis then integrates both the qualitative and quantitative results in order to answer the research question [20, 21]. Ethical approval was granted by La Trobe University Human Research Ethics Committee (ref S17–026). Registered Australian podiatrists were invited via online advertisements to complete an online modified Unified Theory of Acceptance and Use of Technology (UTAUT) questionnaire [22, 23]. The UTAUT theory and associated questionnaire was initially developed and validated to explain psychosocial factors that impact intention to adopt a technology in a commercial context [22]. It has since been further developed and validated in different contexts, including to assess the impact of psychosocial factors impacting technology adoption in the health care settings [23]. Demographic data were collected for all participants, as was key information about their professional practice such as practice type, geographical location and years of practice. The questionnaire was advertised by a number of key Australian podiatry associations and special interest groups including the Australian Podiatry Council, the Advanced Practicing Podiatrists High Risk Foot Group and Diabetic Foot Australia as well as via email to local podiatry networks. The questionnaire was open for 6 months.

Quantitative results derived from the UTAUT questionnaire were analysed to form the basis of the focus group interview schedule. The results were then explored during two focus groups to contextualise and expand on the quantitative findings, while providing participants the opportunity to raise additional factors that would influence their intentions to adopt smart insoles into practice [21].

### Quantitative phase

A modified version of the validated UTAUT questionnaire [23] was used to identify the psychosocial factors that influence podiatrists’ behavioural intention to adopt smart insole technology into clinical practice in Australia. The phrasing used in the UTAUT was modified by substituting the words ‘Henry Ford e-Home Care Telehealth’ with ‘smart insole’ to reflect the technology type being investigated (Table 1 provides example items).

**Table 1:** Example of UTAUT questionnaire

Psychosocial factor	Items	Example item
Performance Expectancy	4	Using smart insole equipment in my practice would allow my patients to be more involved and productive in their health care.
Effort Expectancy	4	I expect learning to operate the smart insole equipment will be easy for me.
Attitude	3	I would like working with smart insole equipment.
Social Influence	4	People who influence my behaviour think that I should use smart insole equipment.
Self-Efficacy	3	I could complete most tasks using smart insole equipment with just the instructions provided me.
Anxiety	4	I worry that if I hit the wrong button my patients’ information may not be collected.
Facilitating Conditions	4	I have the physical and mental ability necessary to use smart insole equipment.
Behavioural Intention	3	If available, I would intend to use smart insole equipment in the next 365 days.

The version of the UTAUT questionnaire used in this study contained seven psychosocial domains and the outcome domain of Behavioural Intention (Table 1) incorporating a total of 29 questions measured on a continuous 5-point Likert Scale ranging from 0 as strongly disagree to 5 as strongly agree with the statement. Where necessary the scores obtained from negatively orientated questions were reversed so that a higher number indicated more support of the underlying attitudes and psychosocial environment for adoption. Mean scores for each domain were calculated. The questionnaire also included four semi open-ended questions, which were analysed by themes and frequency.

In the context of this study, performance expectancy was the degree to which the podiatrist believes that the smart insole will help their patients to self-manage foot monitoring and prevent foot ulceration. Effort expectancy is how easy the podiatrist believes that a smart insole will be to use clinically and issue to the patient. Social influence is the degree to which significant others, such as the patient, other allied health professionals, carers and medical personal, will support the adoption of a smart insole. Self-efficacy is the degree to which the podiatrist believes that they have the skills to adopt a smart insole into their clinical practice. Facilitating conditions refers to the degree to which the podiatrist believes that they have the resources required to adopt a smart insole into their practice. Attitude is the podiatrist’s positive or negative feelings towards using the smart insole with their patients. Anxiety is the self-reported degree of anxiety or hesitation a podiatrist experiences in relation to adopting a smart insole into their practice. Behavioural intention is the podiatrist’s intention to adopt a smart insole into practice within a twelve-month period, if the device were available.

### Statistical methods

All statistical analyses were performed using IBM SPSS Statistics for Windows (Version 25.0; IBM Corporation, NY) and significance was set at *p* < 0.05. Nominal data were presented in proportions, and ratio data were presented as mean and standard deviation. Cronbach’s alpha was used to assess the internal consistency of the UTAUT, with Cronbach’s alpha values being interpreted as follows: α < 0.70 (weak), α = 0.70–0.80 (acceptable), α > 0.80 (strong) [24]. The strength of correlation coefficients were interpreted as follows: *r* = 0.10–0.29 (small), *r* = 0.30–0.49 (medium) and *r* = 0.50–1.0 (large) [25]. Using mean UTAUT domain scores, multivariate regression analysis of the impact of the 7 independent variables on behavioural intention was performed using the Enter method excluding cases pairwise. It was determined that a sample size of at least 49 was required to conduct the multiple regression modelling using 7 independent variables in order to detect a large effect of f^2^ of 0.35 with *p* ≤ 0.05 and power of 80% [26].

### Qualitative phase

#### Focus group

This mixed methods quantitative dominant study sought to obtain sufficient qualitative data to provide context to, and an improved understanding of, quantitative data. Survey respondents were asked to indicate if they were interested in participating in a focus group by ticking ‘yes’ in answer to this question at the end of the questionnaire, and then contacting a researcher to provide their contact details via email. Purposeful sampling was undertaken to ensure podiatrists working in different states and practice settings were represented in the focus groups.

Two focus groups were conducted to explore questionnaire responses. The first focus group recruited *n* = 4 podiatrists from Northern Victoria; *n* = 2 males, *n* = 2 owners of private podiatry practice, *n* = 1 participant employed in mixed private and public practice and *n* = 1 podiatrist who owned a private practice and worked part time in a high-risk foot clinic. Years of podiatry practice ranged from 2 to 12 years. Participants for the second focus group were all attending a national conference to prevent lower limb amputations. Eight podiatrists were recruited for the second focus group, *n* = 6 females. Participants came from around Australia; *n* = 3 from the Northern Territory, *n* = 1 Western Australia, *n* = 2 Tasmania, *n* = 1 New South Wales, *n* = 1 Australian Capital Territory. Participants currently all worked with individuals at high risk of ulceration or amputation in a range of settings; *n* = 2 community-based clinics, *n* = 2 remote clinics, *n* = 4 metropolitan hospital clinics. Four participants also had previous experience working in the private sector. Years of practice ranged from 2 to 21 years.

The focus groups were conducted at Goulburn Valley Health and at the Melbourne Cricket Ground by the first author using a semi-structed question schedule, which prompted exploration of the questionnaire results and group discussion of associated issues. For example, questions included “What do you feel that the barriers would be to using a smart insole in your clinical practice?”

The focus group was audio-recorded and transcribed verbatim using ExpressScribe (5.88, NCH Software, USA). Audio field notes were recorded immediately following the focus groups to allow the researcher to record their reflections and observations from each session [27]. During the conduct of the second focus group the recording was interrupted midway through. In order to address this, at the end of the session, with all participants still present, the moderator recorded a summary of the key points raised. All participants provided their own summaries in their own words. Participants were invited to provide any corrections or clarifications to these summaries prior to the conclusion of the interview.

The transcripts were imported into NVivo for Mac (version 12.6.03841) 1999–2019; QRS International Pty Ltd., Australia) for qualitative thematic analysis. All coding was completed independently by two authors using thematic categories derived from the UTAUT domains. Any data that did not align with the UTAUT domains was coded to describe the meaning of the text, then grouped into categories and then themes that could further explain the quantitative data [27]. Codes were compared and discussed by the researchers to develop the themes, and disagreements were resolved by discussion.

The qualitative data gathered during the two focus groups was sufficient to understand the quantitative questionnaire findings and to address the aims of this QUANT-qual study, indicating thematic saturation [28].

## Results

### Quantitative phase

One hundred and eleven Australian podiatrists completed the online UTAUT questionnaire. The majority of respondents practiced in the private sector (58.6%), were female (50.5%), and held a Bachelor degree (62.2%). Victoria had the single largest group of respondents by state (39.6%) (Table 2). All respondents had access to the internet and telecommunications in their workplace.

**Table 2:** Participant characteristics and UTAUT questionnaire domain mean scores

Participant characteristics	Baseline measurements
Age, years	34.1 ± 9.3
Sex, women	56 (51)
Private practice setting	65 (58.6)
Highest educational level	
Bachelor degree	69 (62)
Years in practice	10.7 (9.4)
Practice States	
Victoria	44 (40)
New South Wales	8 (7)
Queensland	41 (37)
Tasmania	6 (5)
Western Australia	5 (4.5)
Northern Territory	2 (2)
South Australia	3 (3)
UTAUT domain mean scores	
Performance Expectancy	3.20 ± 0.68
Effort Expectancy	3.20 ± 0.67
Attitude	3.27 ± 0.71
Social Influence	2.36 ± 0.85
Self Efficacy	3.01 ± 0.78
Anxiety	0.95 ± 0.85
Facilitating Conditions	3.03 ± 0.66
Behavioural Intentions	3.10 ± 1.00

Data are presented as mean ± SD or number (%)

Internal consistency analysis of the domains of the UTAUT was excellent for behavioural intention (α = 0.98), performance expectancy (α = 0.91), anxiety (α = 0.92), good for self-efficacy (α = 0.90), effort expectancy (α = 0.89), social influence (α = 0.84) and poor for facilitating conditions (α = 0.50).

Significant medium to large positive correlations existed between behavioural intention and six out of the seven independent variables (Table 3). Multiple regression analysis revealed that a single domain, performance expectancy, contributed to the model that was the most predictive of behavioural intention to adopt a smart insole into clinical practice (adjusted R^2^ = 42% *p* < 0.001; Table 3). Social influence was the only other variable that approached significance in this model (β = 0.16, *p* = 0.09).

**Table 3:** Podiatrist UTAUT bivariate and multiple regression analyses with Behavioural Intention

	Bivariate correlation with Behavioural Intention	Multiple regression to predict Behavioural Intention			
Psychosocial domains	(r)	Standardised regression coefficient (β)	Strongest model (β)	Model adjusted R^**2**^	SEE
Performance Expectancy	0.64**	0.42	0.42	0.42**	0.76
Effort Expectancy	0.47**	0.13			
Attitude	0.55**	0.12			
Social Influence	0.45**	0.16			
Self Efficacy	0.30**	−0.30			
Anxiety	0.18	0.02			
Facilitating Conditions	0.36**	−0.06			

*SEE* Standard error of estimate; ^**^p < 0.01.

### Qualitative phase

#### UTAUT questionnaire

Analysis of the open-ended questions found that frequently cited reasons for podiatrists to want to adopt the insole into their practices were that objective feedback to the patient might increase patient knowledge, engagement and self-efficacy (47%) and that the quantitative data would support podiatrists to manage their patients’ foot health (32%). Reasons to not want to adopt the insole included the belief that it would be inappropriate in elderly populations (14%), that the smart insole only monitors the soles of feet (14%), lack of patient access to technology or shoes (11%) and cost (10%). The majority (51.5%) of podiatrists reported either no personal anxiety about using the technology, or no concerns if they were provided with education on usage. However, concerns were raised about the cost of the device to the patient and the device’s utility with the elderly, remote Australian populations and those with active foot ulcerations. Podiatrists also believed that a smart insole would be best targeted towards those with a prior history of plantar foot ulceration (secondary prevention).

#### Focus groups

##### Performance expectancy – “What’s the evidence?” P7b

All focus group participants, regardless of practice setting or state, articulated the need for strong evidence regarding the device’s safety and efficacy in preventing neuropathic foot ulcerations prior to considering adopting a smart insole into practice (Table 4). Without this evidence, all other factors that would impact on their decision to adopt the technology were moot. This is concordant with the regression model developed from the UTAUT questionnaire results, where despite positive bivariate correlations existing for a number of UTAUT domains and behavioural intention, only performance expectancy was predictive of behavioural intention.

**Table 4:** Podiatrist focus group results

Focus Group Theme	Participant Quote
Performance Expectancy	
“What’s the evidence?”	*“I would be open to learning about (a smart insole) if I'm confident and if I've seen the (efficacy) data.” P1a* *“I want to see the evidence behind something that I am giving my patients.” P3b* *“…what's the evidence? What's the benefit to our patients and us as clinicians of incorporating this software? Does it outweigh good diabetic foot education, good footwear education?” P7b*
reliability: “I just want it to work!”	*“I don't want to have to trouble shoot with the device. I want it to just work!” P3b*
“...it is my reputation…”	*“Well if I am using them...it is my reputation...as a clinician (if the device doesn’t perform as expected)...” P1a*
Social Influence	
“…because another patient is going to tell another patient…”	*“If…we had a patient who was willing to tell us about how he used it and how he found it…so we got it from a patient's perspective and then we can say "well actually that's quite good, let’s try it"...You get the patient, then we can get them to talk to...other patients...” P1a* *“a small portion of my clients would be appropriate. And maybe that might have an impact on giving other people the opportunity to witness this and witness the benefits which might…even things up for people who…get written off for things because they're deemed inappropriate from the beginning.” P8b* *“I don't sell anything that I haven’t tried or looked at, or you know someone's recommended...” P1a*
Facilitating Conditions –patient centred	
“…footwear…is going to be a challenge…”	*“…with the variation of footwear we do see… trying to get people to change their footwear, to be able to modify the insole to fit the very wide range of shoes…is going to be a challenge.” P4a* *“I find that a lot of my clients...mobilise in slippers, no shoes at all and in general wear their good shoes down the street and then come home and they won't be in footwear again for the rest of the day.” P2a* *“…some patients (present to clinic)...completely bare foot.” P4b* *“…(a) sock would be preferable to an in-shoe device, and I think that would be particularly pertinent to the private sector where people have to choose between purchasing either multiple devices or being dedicated enough to ensure they have got it on all day.” P3b*
cost: “…biggest barrier is going to be cost”	*“...we were wondering about what the cost might be?” P3b* *“I think the biggest barrier is going to be cost” P3a* *“Cost as well… the towns I service through the public system are very low socioeconomic where they do struggle just to afford a $15 consult fee…I don't think it (smart insole) would be...an option.” P4a*
age: “...your older aged people…would probably find it harder to adapt and learn.”	*“…people in…the younger age bracket will be more inclined…to use the technology as opposed to your quite older aged people, who would probably find it harder to adapt and learn.” P4a* *“But you've got a lot of people set in their ways who will not try no matter what. So I think, especially...the older ones or…the lower social economic people...I think using (a smart insole) would be quite hard for those people.” P1a* *“ You're going to have to direct it to young, middle aged who are going to be tech savvy, who are going to have the watches…. Because anything more complicated you're not going to engage with them, especially the elderly.” P2a* *“A general rule of thumb would be that younger people are more tech savvy and more willing to try and adopt it. In my experience.” P3b* *“…for me, I would base it (recommending a smart insole) on compliance (rather) than age or anything.” P3a* *“...you're gonna have to be selective because those people (not appropriate for insole usage)… not necessarily are elderly. You get a lot of middle aged people who can't get down to their feet...” P4a*
geographical and cultural barriers: “I don't think in Darwin it would work at all!”	*” I don't think in Darwin it (smart insoles) would work at all!” P1b* *“...it’s probably not suitable in an NT indigenous setting due to many barriers including…the fact that many people don't wear shoes or any footwear. Technology is probably something that's.. not really appropriate. People are quite transient, they tend to move around a lot.” P1b* *“ So technology, I mean we're talking about a population base (remote indigenous Australians) that doesn't have mobile phones.” P1b* *And technology itself...the availability and all those things - user access to the technology (to phones, mobile reception electricity)...” P5b*
language and culture: “...people may not understand…”	*“...people may not understand what they're agreeing to, or the requirements that might be involved in that due to the language barrier and may unwittingly agree to something that they don't fully understand..” P1b*
Facilitating Conditions – podiatrist centred	
cost: “...time is money...”	*“...obviously time is money in private. I think in the hospital it wouldn't be an issue, but in private you might have to come up with some solutions to cutting into their time.” P3a* *“getting an item number (for MBS billing for private practice) is gonna be hard as well.” P1a* *“…I'm at capacity already, and this would be another thing that I would need to do...to have time (to) investigate it further, to gain knowledge, and then implement it…would be difficult.” P8b*
“Can it be used in off-loading?”	*“Can it be used in off-loading shoes as well as regular shoes?” P7b* *“..could be used in any off-loading devices such as the CAM boots and Darco shoes with the padding?” P6b*
Target Populations	
secondary prevention:	*“I think in my current setting in the high-risk sector it would be most appropriate for people who have had healed wounds.” P6b* *“I think you'd probably be looking if you get somebody in the 40's or 50's who have diabetic ulcers that'd probably be willing to try...” P1a* *“…those younger people who have had diabetic foot ulcers, that it's had a large impact on their lives. It’s impacted their work, their family life, their social lives and I think they're the ones who'd be very motivated to use this technology because their wounds had such a huge impact on who they are and what they're about.” P3b*
other target populations:	*“...for Charcot transition - so for people who have consolidated their Charcot foot and are returning to weight bearing it would be useful.” P7b* *”I think…the worried well would probably be the best market for this (with) high levels of education.” P6b*

Identifier convention: ‘P’ refers to participant, the numeral denotes the order in which each participant first spoke during the focus group, ‘a’ denotes the regional Victorian focus group and ‘b’ denotes the focus group conducted at a national conference

While the primary motivation for requiring this evidence was for patient clinical outcomes and safety, a secondary element for some private practitioners was linked to the importance of their professional reputations within their communities, and the risk that failure of the device to prevent plantar ulceration would pose to their reputations (Table 4).

##### Social influence “..because another patient is going to tell another patient…” P1a

While not reaching statistical significance in the regression analysis, social influence as it relates to the patient’s perceived willingness and ability to adopt a smart insole was an important factor raised in the focus groups by participants. Participants noted that even if they, as health professionals, believed a smart insole would be beneficial, their patient’s attitudes towards the device, and their physical and financial capacity to adopt the device, would be significant considerations that impact their decision to promote a smart insole to specific patients, or patient groups (Table 4). Some of the factors that might impact on their patient’s willingness to adopt a smart insole related to aspects of the patients’, but not the podiatrists’, facilitating conditions, such as access to and usage of appropriate footwear, their financial situation, their age and access to infrastructure such as electricity and mobile phone reception.

Participants also felt that their patients would be influenced by others’ experiences, either positive or negative, of using a smart insole via ‘word of mouth’. The observation was made that if people with peripheral neuropathy saw others in their social circle successfully using the device, the patient might be more likely to consider using it themselves (Table 4).

##### Facilitating conditions - patient centred (footwear) “..footwear..is going to be a challenge” P1b

Issues related to patient footwear were amongst the most discussed issues across the two focus groups. All podiatrists agreed that they already faced difficulty in convincing patients to wear appropriately fitted shoes with a fastening (Table 4). This difficulty was most pronounced in very hot parts of Australia where the majority of people wore thongs or sandals, and where many people, particularly indigenous Australians, commonly walked barefoot (Table 4). As the smart insole described to participants was required to be worn in an appropriately wide shoe, with a fastening such as laces or velcro, podiatrists believed that this would limit the number of people suitable for the device. Furthermore, podiatrists working with people with active foot ulcerations felt that the device would not be suitable if it could not be used with off-loading modalities such as wound shoes, controlled ankle motion (CAM) boots or custom-made foot orthoses (Table 4).

Some podiatrists believed that a more flexible device in the form of a textile, such as a smart sock, would be more useful as it could be used in a wider variety of footwear, or no footwear at all.

##### Facilitating conditions - patient centred (cost) “I think the biggest barrier is going to be cost.” P3a

The likely cost of a smart insole to the patient was a consistent concern for both public and private sector podiatrists from across Australia in the focus groups, as it was in the questionnaire responses. Podiatrists reported that many of their patients already struggled with the cost of purchasing appropriate footwear, and that unless the device was subsidised it would likely be beyond the reach of patients from lower socioeconomic backgrounds (Table 4).

##### Facilitating conditions - patient centred (patient age) “..your older aged person..would probably find it harder to adapt and learn.” P4a

There was a majority view amongst focus group participants that patient age might be a barrier to adopting smart insoles, and that smart insoles would be best targeted towards younger and middle-aged patients who are comfortable with utilising other technologies (Table 4). While there was some nuance in the discussion, with recognition by one participant that younger people can have physical limitations that might make insole usage difficult, and other participants who acknowledged there were tech-savvy elderly, some participants still firmly believed that patient age in and of itself would be a barrier to adoption (Table 4).

##### Facilitating conditions - patient centred (geographical and culture barriers) “I don’t think it would work in Darwin!” P1b

An important factor for podiatrists servicing more remote locations in Australia, such as central and far northern Australia, as well as those servicing patients from disadvantaged backgrounds, was access to appropriate technology infrastructure to support the use of an electronic monitoring system. Podiatrists reported that in some remote communities, there is not consistent access to electricity or electrical outlets at which to charge devices, and there is no network coverage for the internet or mobile phones. Therefore, electronic monitoring systems, such as a smart insole, could not reliably be utilised in these communities (Table 4).

Participants working with patients from non-English speaking and Aboriginal and Torres Strait Islander backgrounds had some concern about the cultural appropriateness of a smart insole due to attitudes to footwear usage, as well as language and cultural barriers impacting the capacity of patients to understand what was involved in using a smart insole prior to agreeing to it being issued (Table 4). In this context, they believed that additional care would need to be taken in patient-selection and ensuring careful, clear communication.

##### Facilitating conditions- podiatrist centred (cost) “..time is money..” P3a

Another aspect related to the ‘cost’ of using a smart insole in podiatry practice was a concern about how long it would take for the podiatrist to learn how to use and issue such a device, and to support patients in adopting and using it. Some podiatrists in private practice reported that ‘time is money’ and therefore anything that was time intensive to issue would be less attractive in this setting (Table 4). While the majority of podiatrists from public practice did not feel that the time taken for them to issue a smart insole to a patient would be a barrier to adoption, there was one podiatrist that reported they were time poor due to very high demand on the service, and felt that adopting smart insoles into their practice would be an additional time burden they could not afford (Table 4).

##### Facilitating conditions- podiatrist centred (compatibility with off-loading) “Can it be used in off-loading?” P7b

A significant issue for podiatrists working with patients with active foot wounds or Charcot’s neuroarthropathy was the need for any smart foot monitoring device to be compatible with evidence-based off-loading commonly used for ulcer prevention and management, including medical grade footwear, custom made foot orthoses, podiatry felt padding, CAM boots and wound shoes (Table 4). A device that is incompatible with these modalities was seen as less beneficial in a high-risk foot setting.

##### Target population – secondary prevention

There was agreement across focus groups that the most appropriate target population for a smart insole would be those utilising it for secondary prevention of neuropathic plantar ulcers, with use following consolidation of Charcot’s neuroarthropathy also suggested (Table 4). The belief that secondary prevention should be targeted was due to a number of factors, including that people who have experienced a serious foot pathology first hand will be more motivated to monitor their feet, and be most likely to wear shoes compatible with a smart insole (Table 4).

One participant did note that there might be a niche target group amongst well educated, tech savvy individuals whom they deemed the ‘worried well’ who might be more likely than average to adopt smart insole technology before they had experienced a primary foot ulcer (Table 4).

## Discussion

Podiatrists who participated in this study were open to smart insole technology adoption, but first required sufficient evidence of clinical efficacy in the proposed target population. This key finding was highlighted by the fact that while significant positive correlations existed between performance expectancy, effort expectancy, attitude, social influence, facilitating conditions, self-efficacy and the outcome variable of behavioural intention, performance expectancy alone explained 42% of the variance in intention to adopt smart insoles. The importance of performance expectancy was further highlighted in the focus groups during discussions of clinical efficacy, and by questionnaire semi open-ended responses where improvements in patient foot self-management and knowledge were the most commonly cited reasons to adopt a smart insole into clinical practice.

The requirement to have strong supporting clinical evidence of safety and efficacy prior to adopting a new modality into clinical practice is congruent with the overarching ethical requirements of podiatrists to act in their patient’s best interests, using evidence-based medicine [29]. In the case of a commercially available smart insole, evidence of efficacy in ulcer prevention remains uncertain [10, 30, 31], despite a number of reports that provide encouraging results for its use in secondary prevention [16, 32–34]. Given this, further research including larger sample sizes which provide robust evidence of efficacy in primary or secondary foot ulcer prevention is required in order for podiatrists participating in this study to consider broadly adopting smart insole technology.

The qualitative data, in addition to confirming the importance of performance expectancy to podiatrists, provided a more nuanced understanding of the secondary issues impacting their adoption intentions and highlighted barriers that would need to be overcome to facilitate adoption. It is noteworthy that many of the secondary issues identified in the qualitative data were patient related, and that in the quantitative model social influence, of which patient attitude is a key element, approached significance in the regression model. Some of the secondary issues identified by podiatrists echoed concerns raised in the patient study [18], indicating some common barriers that would need to be addressed to encourage smart insole adoption for both adults with diabetes and podiatrists. Shared potential barriers included concerns around smart insole compatibility with patient footwear and orthotics as well as the cost of the device. Other barriers raised by podiatrists, including patient footwear access and usage, patient age, geographical location, and compatibility with acute pressure offloading modalities, were concerns for the podiatrists, but not for the patients themselves.

The issue of patient access to, and use of, appropriately fitted footwear suitable for use with a smart insole was consistently raised by participants in the focus groups, and to a lesser extent in questionnaire responses, as a likely barrier to broad adoption by their patients. In Australia medical grade footwear and custom made orthoses for people at high risk of ulceration are not consistently publicly funded despite evidence of efficacy and cost effectiveness for foot ulcer prevention, and recommendations by peak Australian bodies for federal government funding [6]. Therefore, cost acts as a barrier to access to appropriate footwear for some Australian populations [7]. In addition, as discussed by focus group participants, there are large geographical variations in climate in Australia, and cultural differences in attitudes towards footwear, with populations living in hot areas, and those from indigenous backgrounds less inclined to wear footwear and off-loading that meets the Australian guidelines for footwear for people with diabetes [6]. Outside these particular climatic and cultural challenges, ensuring patients within the broader diabetes population at high risk of developing foot ulcerations adhere to currently prescribed footwear and pressure off-loading modalities is already an ongoing challenge for health professionals [35–37]. Binning et al. [35] found that patient adherence to protective foot care behaviours following ‘traditional’ patient education was poor, and Perrin et al. [38] found that despite participants reporting high levels of self-efficacy in regards to enacting footcare recommendations, actual foot care behaviours often diverged from recommendations for a range of reasons [38].

As highlighted by participants in this study, it is likely that podiatrists would face similar challenges in encouraging people with neuropathy to adopt and consistently wear a smart insole, as it will only be used as consistently as the person wears the shoes in which the insole rests. One approach, which has demonstrated promise in improving patient adherence to footwear use in the short term, is motivational interviewing [35]. In order to increase the likelihood of smart insole adoption, alternate approaches to increasing patient adherence to footcare recommendations, such as motivational interviewing, should be investigated in a clinical context. An alternative solution raised in the focus groups was to use a device with sensors embedded into a textile, such as a smart sock, that would be able to monitor the feet in any pair of shoes, or even when the patient did not wear shoes. Internationally, research involving sensors embedded into textiles that measure a range of factors, including temperature and pressure [12–14], step number and velocity [39] is occurring and holds the promise of being able to fill different clinical foot monitoring requirements in the future.

Concerns regarding the cost of a smart insole also weighed heavily on podiatrists in focus group discussions and questionnaire responses when considering the suitability of the device in their own practices. Many podiatrists working in public practice, high-risk foot clinics and private practice, felt that as with medical grade footwear, unless the devices were subsidised it would be beyond the reach of many of their patients. This echoes the concerns raised by regional adults with diabetes, who also identified device cost as a likely barrier to adoption, and raised the possibility of government funding to support its use [18]. If, in future, convincing evidence of efficacy in preventing foot ulceration occurrence was demonstrated, a cost effectiveness evaluation should be undertaken in the Australian context to determine if government funding for wearable foot monitoring technology for people at high risk of foot ulceration is warranted. A cost effectiveness study that modelled the use an existing smart insole, the SurroSense Rx, as an adjunct to standard care for people at risk of recurrence of diabetes associated foot ulceration has already been conducted. The results of this study demonstrated such a device could be cost effective, as long as efficacy data obtained from a small cohort trial were confirmed by larger scale, multisite longitudinal studies [40].

The belief that this type of device would be most suited for secondary prevention of foot ulcers was evident in both the questionnaire responses and focus group discussions, and is consistent with risk stratification principles targeting additional preventative resources to those at highest risk of foot ulceration [2, 40]. Participants in this study felt that this cohort were more likely to be motivated to prevent a recurrent foot ulcer by their previous experiences. This perception is supported by research regarding cognitive representations of people with neuropathy which found that those with previous history of DFD reported engaging in less damaging foot behaviours and having a more realistic understanding of their condition [41]. Given that the recurrence rate of foot ulcers is estimated to be 40% within the first 12 months post wound healing [3], and that the incidence of both diabetes and foot ulceration and amputation are increasing in Australia [2], secondary prevention represents a sizable market and an opportunity to save significant health care resources. While use of a smart insole in secondary prevention is important and might represent the main target group, serious consideration should still be given to utilising this type of device for primary prevention in populations with multiple risk factors and who are amendable to adoption.

Patient age was identified as a barrier to adoption of a smart insole by many participants, who felt that middle aged, tech-savvy patients would be a better target for this modality. However, this view is not supported by the results from a previous study of patients with a mean age of 62 years, which revealed that these older participants had a very positive attitude and reported high levels of self-efficacy towards the concept of adopting a smart insole [18]. The vast majority of these older participants also had internet access, which was reflective of the high rate of uptake of telecommunication devices such as smart phones in the broader Australian community, including older Australians [18, 42]. Similarly, a recent meta-analysis found that variables, such as perceived threat of poor health, perceived ease of use and trust influence older users adoption of mobile health technology in order to maintain their health [43]. Therefore, chronological age should not deter podiatrists or other health professionals from offering a patient the option of using emerging health monitoring technology. Instead, each patient should be individually assessed, and their particular circumstances considered, so that a tailored plan can be developed that best meets the needs of the individual [29].

### Limitations

A limitation of this study was that because it sought to explain behavioural intention, rather than actual behaviour, participants did not use a specific technology and therefore were not able to evaluate device specifications and functionality to inform their responses. Therefore, results should be interpreted as attitudinal intention towards adopting a generic form of ‘smart insole’ into future practice, rather than adoption intentions towards a specific device. Additionally, those who chose to respond to the online smart insole adoption questionnaire might have strong feelings towards this form of technology not held by those who chose not to respond, limiting generalisability of the results. Despite this, a cross section of respondents from various states of Australia, working in a variety of clinical practice settings, providing a range of perspectives on device adoption in the Australian podiatric context were included.

## Conclusions

Australian podiatrists were open to adopting smart insoles into clinical practice, however they first require strong evidence of the device’s clinical efficacy. Other barriers to usage, such as device cost, access to and usage of appropriate patient footwear, compatibility with therapeutic off-loading modalities, and podiatrists’ perceptions towards patient age acting as a barrier, would then need to be overcome in order for a smart insole to be adopted by Australian podiatrists into clinical practice.

## Data Availability

The datasets generated and analysed during the current study are available from the corresponding author on reasonable request.

## Abbreviations

DFD: Diabetic Foot Disease
DFU: Diabetic Foot Ulcer
UTAUT: Unified Theory of Acceptance and Use of Technology
CAM: Controlled Ankle Motion
